# Females sample more males at high nesting densities, but ultimately obtain less attractive mates

**DOI:** 10.1186/s12862-015-0481-3

**Published:** 2015-09-18

**Authors:** Robin M. Tinghitella, Chelsea Stehle, Janette W. Boughman

**Affiliations:** Department of Biological Sciences, University of Denver, 2190 E Iliff Ave., Denver, CO 80210 USA; School of Biological Sciences, University of Nebraska, 1044 T Street, Lincoln, NE 68588 USA; Department of Integrative Biology and BEACON Center for the Study of Evolution in Action, Michigan State University, 288 Farm Lane, East Lansing, MI 48824 USA

## Abstract

**Background:**

Sexual selection is largely driven by the availability of mates. Theory predicts that male competition and female choice should be density-dependent, with males competing more intensely at relatively high density, and females becoming increasingly discriminating when there are more males from whom to choose. Evidence for flexible mating decisions is growing, but we do not understand *how* environmental variation is incorporated into mate sampling strategies. We mimicked threespine stickleback (*Gasterosteus aculeatus*) breeding conditions in pools with high and low densities of nesting males and allowed females to search for mates to determine whether 1) mate search strategies change with the density of breeding males and 2) pre-copulatory components of mate choice (signalling, competition, search patterns, and mating decisions) are modified in parallel.

**Results:**

While females sampled more males at high male density, suggesting greater opportunity for sexual selection, the expanded search did not result in females choosing males with more attractive sexual signals. This is likely because red throat colouration was twice as great when half as many males competed. Instead, females chose similarly at high and low male density, using a relative strategy to compare male traits amongst potential suitors. Reduced throat colour could reflect a trade-off with costly male competition. However, we did not observe more intense competition at higher relative density. Density-dependent signalling appears largely responsible for females associating with males who have more attractive signals at low density. If we lacked knowledge of plasticity in signalling, we might have concluded that females are more discriminating at low male density.

**Conclusions:**

To understand interactions between mate choice and population dynamics, we should consider how components of mate choice that precede the mating decision interact.

**Electronic supplementary material:**

The online version of this article (doi:10.1186/s12862-015-0481-3) contains supplementary material, which is available to authorized users.

## Background

Mating decisions were traditionally viewed as relatively static within individuals [1, 2] but a growing body of work demonstrates instead that they are highly dynamic, changing in response to factors intrinsic to females as well as to experience and prevailing ecological conditions (e.g. [3–11]). This emphasis on plasticity in mate choice parallels evolutionary ecologists’ commitment over the last 30 years to discovering which male characteristics are favoured by females and why. However, the question of how females sample and compare mates has historically received less attention. Similarly, our knowledge of *how* females incorporate environmental variation into the search for mates lags behind the knowledge that they can and do alter decisions [12, 13]. This is despite the fact that different search strategies can lead to different mate choices, which means search strategies have important implications for the strength of sexual selection and the speed with which correlations develop between the genes for male traits and female preferences [12, 14–16]. We focus here on how mate availability, a key variable generating differential sexual selection, impacts male and female behaviour during the search for mates.

Numbers of potential mates can vary in two ways: biased sex ratios increase the mate encounter rate for one sex, and variation in population density alters mate availability in the same direction for both sexes [15]. Classic sexual selection theory predicts that as the ratio of available males to females (the operational sex ratio; OSR) increases, male competition for reproductive resources and access to females also increases ([17], reviewed in [18]; but see [19] and [20] for additional discussion of this hypothesis). Furthermore, the proportion of males unsuccessful in male-male competition and female choice is typically highest at high male density (individuals per unit area; [15]). Theory also predicts that when males are readily available, either because of a male-biased sex ratio or high population densities, females will adjust their choices to reflect search costs and can afford to be more selective [15, 21–23]. Although these predictions may be overly simplistic, we nevertheless expect the evolution of reaction norms that allow individuals to adjust their mating behaviour to reflect the prevailing availability of mates [15, 20, 24]. This should be particularly likely when mate availability varies on short timescales [25]. Existing empirical work in a variety of systems supports the predictions that intraspecific competition should be greater at higher overall densities and that females are more discriminating when males are common (e.g. [5, 7, 10, 26–28]). This is not the case for all study systems, however. For instance, both territoriality and female choice can break down at particularly high male densities because the cost of competition may also increase (e.g. [29–32]).

How might the availability of males impact the consequences of female mate sampling strategies specifically? Environmental constraints on the timing of reproduction and predation risk limit the time females have in which to mate. Consequently, females usually do not sample all available males, and selection should favour mate search strategies that balance the benefits of finding a preferred mate and the costs of extended sampling. There are two commonly considered categories of mate sampling strategies: relative and absolute (also called threshold; [33–36]). Relative strategies involve comparing the traits of males who are sampled. A female’s preference is relative if she selects a male with a more developed form of the secondary sex character in a given group, regardless of the group’s position along the population-level distribution of the trait [14]. For instance, a female pursuing a ‘best of N’ strategy might sample a number (N) of males and then return to the highest quality male [33]. Females using absolute or threshold strategies, on the other hand, compare the traits of sampled males to an internal standard and continue to search until they encounter a male whose trait exceeds the threshold value [33]. These general models have been criticized for being too simplistic, and more recent work builds on them by incorporating complexity like search costs and limits to information processing and memory (e.g. [36–40]). For instance, flexible strategies, like the variable threshold strategy [33, 41] allow for acceptance thresholds to change based on sampling costs. Empirical work aimed at determining precisely how females sample mates is scarce [3, 12] and the same sampling pattern can suggest more than one strategy [42], so it can be difficult to make straightforward predictions about how mate availability should influence the search for mates. Two key variables, however, are the number of mates sampled, and the initiation of courtship by sampling females [12]. Here, we concentrate on whether these aspects of the female search depend on the density of nesting males, the consequences of mate sampling for mating skew (the proportion of males that do not mate), and the quality of chosen males.

If the behaviour of females does not change with density, we expect females to sample the same number of males (relative strategies) or maintain the same internal standard (absolute strategies) across densities. At low male density (a female-biased sex ratio or overall low density), a fixed strategy should increase search costs (distance, time), altering female choice by reducing the probability of being mated and/or the ability to find the most preferred mates. In either case, the opportunity for sexual selection will be greater at high density. Alternatively, if mate choice rules change and females are less discriminating when mates are rare (either because they search through fewer males or the threshold of acceptability is lower), sexual selection is relatively relaxed [15, 23, 43]. Flexibility in the mate search may help individuals to buffer the risk of not mating at very low densities, mitigate mate finding Allee effects (the difficulty of finding mates at low densities; [15, 23, 44]), and reduce the risk of population extinction when mates are limited or costly to find [45, 46].

Although most mate-sampling work places the emphasis on the searching female (but see [12, 47]), the search for mates does not occur in a vacuum. Several pre-copulatory components of choice influence mating decisions, including sexual signalling, competition for territories and nest sites, female sampling strategies, and courtship by males [48]. All of these may change in step with ecological conditions. Thus, a realistic understanding of mating dynamics and sexual selection must include the impacts of mate availability on multiple components of choice [13], which may or may not be modified in parallel by experience (e.g. [49]). Sexual signalling strategies, for instance, could dramatically alter female mating decisions. Male competition changes the expression of traits that females use as cues during mate choice, including in our study system, the threespine stickleback (*Gasterosteus aculeatus*) [50]. If male competition alters signalling, the distribution of available signals and the frequency with which females encounter them could lead to different female mating decisions at high vs. low breeding male density, blurring the lines between the contributions of mate availability and mate quality to density-dependent mating decisions. Similarly, when competition for access to females varies, males may alter the frequency and vigour with which they court particular females, particularly in mutual mate choice systems. Finally, females are not passive courtship recipients, and may initiate courtship themselves [12].

Here, we specifically consider how sexual signalling, competition for territories and nest sites, and courtship impact female search strategies and mating decisions across an established range of nesting male densities commonly experienced by British Columbian *G. aculeatus*. Our experimental design mimics a female arriving on the breeding grounds for the first time, assessing male availability and signal quality, and searching amongst nesting males. We varied male density at a 3:1 male:female adult sex ratio in order to obtain an OSR (nesting males:gravid females) close to 1:1. This was because we expected for some, but not all males to nest in our treatment pools. The low male density treatment contained three potential male competitors and the high male density treatment contained six potential male competitors. We hypothesized generally that precopulatory stages of mate choice would be modified in parallel [49] across nesting male densities. We first asked whether aspects of males’ sexual signals varied with the density of competitors. *G. aculeatus* males with brighter red throats enjoy higher dominance status [51], have an increased tendency to attack other males [52, 53], and are less frequently attacked by competitors [54–57], so we expected males to increase effort placed in signalling under high density conditions. Alternatively, if competition enforces the honesty with which individuals signal, as has been observed in other populations of sticklebacks, males may instead reduce the effort placed in signalling or not be able to produce high intensity signals because of trade-offs with costly competition at high density [50]. Second, we assessed male competition, testing the well-substantiated hypothesis that at high male density males engage in more competition (e.g. [15, 28]). We tested a) whether there are more competitors (nesting males) in the mating “pool”, b) if males engage in more competition and/or escalated fights, and c) if males physically compete with more individuals at high density. Finally, we turned our attention to female search strategies and mating decisions, testing the hypotheses that a) female search strategies are plastic, and b) females are more discriminating when there are more males from whom to choose. *G. aculeatus* females evaluate multiple males before laying a clutch of eggs, and previous work demonstrated that females in some populations (including British Columbian lakes) use sequential mate choice strategies [58]. If female search strategies are density-dependent and sequential, then at high densities we expected them to sample more males and/or to show reduced interest in males with lower quality sexual signals if using a variable threshold search strategy [33, 41]. If a fixed threshold strategy is in place, the acceptance of males with a given sexual signal should not depend on search costs or density.

In short, we found that female search strategies are density-dependent and depend heavily on plastic male signalling strategies. Females used a relative search strategy at both densities, choosing males whose sexual signals ranked above the mean relative to competing courters. Ultimately, this meant that females associated with more attractive males at low, not high densities.

## Methods

### Study species

British Columbian *G. aculeatus* experience different levels of mate availability within their lifetimes [11]. Early in the breeding season, the density of reproductively ready limnetic males caught in minnow traps on the breeding grounds is 16 times higher than it is late in the season (RM Tinghitella and JW Boughman, unpublished data). The density of gravid females also declines precipitously – there are 14 times fewer gravid females on breeding grounds late in the season. It is unknown what causes the dramatic reduction in population density over the season, although a combination of predation and the timing of reproduction may be responsible. Male sticklebacks arrive on the breeding grounds days to weeks before females and compete strongly with one another to establish territories and build nests. Competition is direct and also mediated through sexual signals, which mitigate the costs of competition [54–57]. After the competition phase, females arrive and search amongst courting and nesting males to determine with whom they will spawn. Mating decisions are based on assessment of complex male sexual signals; the most important is the red throat, a carotenoid based signal that contrasts strongly with males’ blue body and blue eye colouration [59, 60]. The female preference for redder males is well-established, and females who spawn with more intensely coloured males gain direct and indirect benefits including quality parental care, and high condition, parasite-free mates [55, 61–63].

In April of 2012 we collected reproductively ready limnetic males and females from Paxton Lake on Texada Island, British Columbia and transferred them to Michigan State University (MSU). There, we housed them in 110-L tanks (approximately 77 × 32 × 48 cm), separated by sex at a density of ~20 fish/tank. The tanks were in a temperature and photoperiod controlled room set to 17 °C and 15:9 h light:dark. One to two days prior to being used in behavioural trials, males were transferred to a satellite outdoor facility on campus. At the satellite facility, fish were housed in identical 110-L tanks, but lighting was natural (MSU: 42.78°N, 84.48°W; Texada Island: 49.67°N, 124.42°W). We kept females at the indoor facility until they showed signs of becoming gravid and transferred them when we expected them to reach full gravidity within 24 h. In both settings, fish ate a diet of *ad libitum* bloodworms and brine shrimp daily. Behavioural trials took place in June and July of 2012.

We conducted behavioural trials in hard plastic round pools (1.52 m in diameter and filled to 0.25 m; 463.4 l) with bricks and artificial plants for cover, and a sandy bottom. Pools were housed outdoors under shade provided by nylon tents and covered individually with mesh to prevent predation. We varied density at a 3:1 male:female adult sex ratio in order to obtain an OSR (nesting males:gravid females) close to 1:1. We first randomly assigned three males to each low density replicate pool and six males to each high density replicate pool, approximating the variation observed on Paxton Lake breeding grounds (RMT and JWB, unpublished). OSRs obtained are reported in the results. All males were reproductively ready as indicated by breeding colouration. 24 h after being placed in a pool, we marked males with a unique coloured elastomer tag (Northwest Marine Technology Inc.). To encourage males to nest, we provided natural nesting material and exposed males to a gravid female for 15 min daily (in a clear glass jar) until at least one male in the pool had nested. We checked pools daily for evidence of nest building and considered a nest complete when it had an entry hole and an exit hole and the nest owner was observed going completely through the nest. Males were able to freely choose nesting locations anywhere along the sand-covered bottom of the pool. We also measured fish length to the nearest 0.01 mm using digital calipers to ensure fish size did not differ between density treatments. It did not (females: low = 42.07 ± 1.02 mm, high = 43.07 ± 1.05 mm, t = 0.684, *p* = 0.50; males: low = 46.08 ± 0.65 mm, high = 45.99 ± 0.47, t = 0.11, *p* = 0.91).

### Sexual signalling

To assess whether the sexual signals of males kept at alternative densities differed, we took colour measurements on each male immediately after each set of behavioural trials. Colour measurements followed a standardized colour scoring method [11, 64] that closely matches reflectance data [65] and minimizes handling time. We dip netted each male from the pool, in random order, held them in hand with the right lateral side of the body facing up, and recorded five components of colour: the area expressing red, the intensity of red, the intensity of eye colour, the intensity of body colour, and overall body darkness. Each colour component was scored on a scale from 0 to 5 where 0 represented no colour and five represented maximum colour or intensity. To establish a males’ score, he was compared to a set of photo standards for each component. The standards were amassed over 15 years of work with these fish and each photo differs by a score of 0.5 points for a given colour component, allowing us to assess color more objectively. We calculated a red throat index for each male as the sum of throat area and throat intensity (a maximum score of 10).

### Male competition, female experience, and search and choose trials

After males established territories and built nests, we conducted three phases of behavioural trials: *Focal Follows* of each male, *Female Experience* trials, and *Female Search and Choose* trials. Focal follows documented whether and how competition interactions vary when competitors are rare vs. common. We first introduced a focal, gravid female to a 20 cm diameter clear glass cylinder in the center of the pool. To keep the adult sex ratio equal across density treatments (3:1 male:female), we also introduced a second, non-focal gravid female to a second glass cylinder in the high density pools. In the low density pools, a second empty glass cylinder was introduced. We then performed a 5-min focal follow on each male present in the pool in random order, recording the number and type of all male-male competition behaviours (Additional file 1) directed toward the focal male and performed by the focal male toward others in the pool. Behavioural observations were recorded using JWatcher v 1.0 [66].

Following the *Focal Follows*, we turned our attention to the experience that the focal female had at high vs. low density during a 15-min long *Female Experience* trial. The focal female remained in the glass cylinder during these trials because they were designed to assure that the female interacted with each of the males present in the pool, allowing her to assess mate availability. We recorded all courtship behaviours directed toward the focal female as well as the male-male competitive interactions that occurred directly outside of the female’s glass cylinder in her field of view. We determined how many males courted the female and how many male competition behaviours were observed. Because male competition and courtship consist of a series of graded behaviours, we also assessed the magnitude of competition and courtship (scales used to assess magnitude of behaviors are described below).

Finally, we released the female from her glass cylinder for a 30 min *Search and Choose* trial during which she interacted freely with all males present in the pool. To prevent female-female competition from interfering with the focal female’s mate search, we removed the non-focal female from the glass cylinder in the high density pools immediately before releasing the focal female. While we recognize that female-female competition may also change with mate availability, we were particularly interested in documenting plasticity in search strategies in this experiment, not female competition. We again recorded all behavioural interactions between the focal female and males in the pool. Behaviours recorded during *Search and Choose* trials were modified to indicate the identity of the interacting male so that we could determine to whom the female directed interest during courtship interactions. Two observers participated in *Search and Choose* trials; one verbally announced behaviours and identities of actors, and a second operated the event recorder. In all replicates, the female interacted with each male present in the pool prior to the *Search and Choose* trial (during either male *Focal Follows*, *Female Experience* trials, or both).

We ran 21 high density sets of trials (*Focal Follows*, *Female Experience*, and *Search and Choose* trials). Two high density sets of trials were eliminated because the females were not courted. This left 19 *Search and Choose* trials. Of those, we have *Female Experience* trials for all but one, because we did not begin recording female experience until about a week into the experiment. We also ran 21 low density sets of trials. Three of these were eliminated because the female was not courted. Of the remaining 18, we have *Female Experience* trials for all but two.

### Statistical analyses

Throughout the analyses, non-significant interaction terms were removed from final models in a stepwise manner. We used mixed modeling (Restricted maximum likelihood (REML) mixed models and Generalized Estimating Equations (GEE)) because a given set of males (in a replicate pool) was used between one and three times with different gravid focal females on different days, although most replicate pools (and their set of males) were used only once. Eleven replicate pools were used once, seven were used twice, and four were used three times. Replicate pools were reused with multiple females because the process of establishing a new replicate and allowing males to nest took up to two weeks. These fish are also from protected populations, so we made every effort to minimize the number of individuals utilized. Females were not reused. All REML mixed models were performed in JMP v. 11. Generalized Estimating Equations, which extend the mixed model framework to categorical outcome variables, were performed in SPSS v. 20. Unless otherwise described, the basic model structure for male signaling and competition measures assessed the explanatory variables male density (high or low), day of season, nesting status, and their 2- and 3-way interactions. Replicate pool was a random effect in the male models. Nesting status was included because red throat colour may depend on nest establishment. The basic structure of models assessing measures of female experience, search strategies, and choice of males included the explanatory variables male density (high or low), day of season, and their interaction. Female ID nested within replicate pool was the random effect. All means reported are LS means ± 1 SE.

#### Sexual signalling

We used REML mixed models to assess differences in the dependent sexual signalling variables red throat index (area + intensity), body colour, eye colour, and body darkness. Sexual signalling peaks mid-season in this population ([11] and this study), so we included a quadratic day of season term to test for a nonlinear effect of age and a nonlinear interaction between day of season and density. Each signal component was assessed in a separate model.

#### Male focal follows

We used both GEEs and REML mixed models to assess male competition. In addition to the basic structure reported above, we included red throat index as a covariate because throat colour varied with both male density and time of season (see *Results*), and influences the direction and severity of male competition [54–57]. The dependent variables in the REML mixed models were the sum number of aggressive behaviours given and received, the escalation of those behaviours (on a competition scale of increasing aggression ranging from 0 to 4 where 0 = no interaction and 4 = mouth wrestling), and the number of males competing with the focal male. The dependent variables in the GEEs were whether the male received aggression from non-focal males, and whether or not focal males aggressed against non-focal males.

#### Female experience

Dependent variables in the female experience REML mixed models were the sum number of courtship behaviours directed toward the focal female, the number of males courting the female, and the sum number of male-male competition behaviours observed by the focal female. The number of male competition behaviours observed was square root transformed to improve normality prior to modeling.

#### Female search and choose

The nature of the *Search and Choose* trials allowed us to observe the full complement of courtship and competition behaviours (Additional file 1). In addition to the three dependent variables assessed in the *Female Experience* trials, we also considered responsiveness, acceptance score, and female initiation of courtship (approaching non-courting males, visiting their nests outside of active courtship, or angling; Additional file 1). Responsiveness is a measure of motivation to mate (the number of times a female followed a male when he led her to the nest), and acceptance score measures how far courtship progresses (ranging from no response to attempted spawning on a scale of 0–4; ‘preference’ score in [11, 67]. Because some *Search and Choose* trials ended when the female entered the nest rather than after the full 30-min observation, when the number of a particular type of behaviours was assessed, we considered behaviours/minute. Two final models assessed the key characteristics of the chosen males. The dependent variables were the red throat index of the “chosen” male (the male with whom the female proceeded furthest in courtship) and his courtship vigour (courtship behaviours per minute).

## Results

### Nesting and sexual signalling

**Fig. 1:**
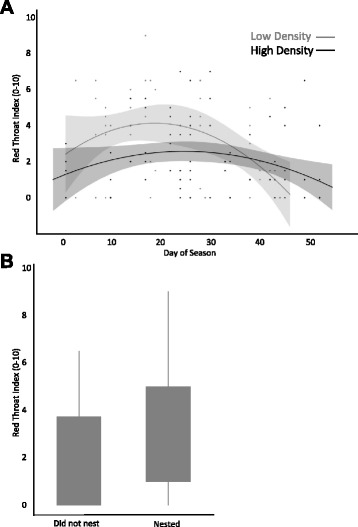
Throat indices across male densities and among males who did and did not nest. **a** Red throat index (0–10) of males in low male density and high male density pools across the breeding season. Represented are the quadratic regression lines and confidence intervals (*shadows*). **b** Red throat index of males who did and did not establish a territory and build a nest. Boxes enclose the interquartile range (IQR) and horizontal bars represent the median. Whiskers extend to include the furthest point within 1.5x IQR from the box

Of the colour variables measured, only the red throat index (area + intensity) was associated with treatment density, whether a male nested, and/or the time of season (Table 1). Red throat index ranged from 0 to 9 on a ten-point scale. Males in the low male density treatment attained a red throat index more than two times greater than those in the high male density treatment, and throat indices peaked in mid-season (Fig. 1a). There was a significant interaction between time of season and treatment on red throat index, indicating that the pattern of change over the season varies across male densities. Namely, the mid-season peak in throat colouration was more pronounced under low than high male density conditions (Fig. 1a). Importantly, at both high and low male densities, males who nested had greater red throat indices (Fig. 1b).

**Table 1:** REML Mixed Models of factors affecting four aspects of male colour: red throat index, eye intensity, body intensity, and body darkness

Colour measurement	Fixed effect	*F*	*df*	*P*
Red Throat Index	**Male Density**	**7.61**	**1, 18.28**	**0.01**
	**Nesting Status**	**5.40**	**1, 126.8**	**0.02**
	**Quadratic Day of Season**	**7.54**	**1, 15.85**	**0.01**
	**Quadratic Day of Season x Male Density**	**4.62**	**1, 16.14**	**<0.05**
Eye Intensity	Male Density	0.01	1, 16.80	0.92
	Nesting Status	1.53	1, 126.4	0.22
	Day of Season	2.87	1, 19.14	0.12
Body Intensity	Male Density	1.76	1, 15.00	0.20
	Nesting Status	2.48	1, 125.8	0.12
	Day of Season	0.51	1, 17.35	0.48
Body Darkness	Male Density	0.84	1, 15.07	0.38
	Nesting Status	3.78	1, 122.9	>0.05
	Day of Season	1.81	1, 17.69	0.20

Replicate pool was a random effect in all models and significant results are indicated in bold

**Fig. 2:**
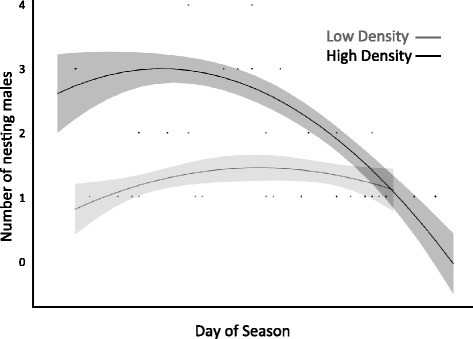
Number of males nesting in high male density and low male density pools across the breeding season. Represented are the quadratic regression lines and confidence intervals (*shadows*)

Male density was an important predictor of the number of nesting males. On average 1.23 ± 0.22 males (range 1–2) nested in low male density pools and 2.32 ± 0.24 males (range 1–4) nested in high male density pools (Fig. 2; F_1,18.65_ = 11.35, *p* < 0.01). This makes the realized OSR in the low male density pools 1.23:1 (males : females) and in the high male density pools 1.16:1. Approximately 40 % of males nested overall in each treatment. Thus, the realized density of nesting males was 89 % greater in the high male density treatment than the low male density treatment. The number of males nesting did not vary seasonally (F_1, 40.05_ = 1.54, *p* = 0.22).

### Male competition

We observed 7.15 ± 0.60 competition behaviours (charges, bites, chases, mouth wrestles, and nest destruction) during focal follows (range 0–31). Wrestles and nest destruction were rare, occurring in only 5 of 137 and 1 of 137 focal follows, respectively. Some aspects of male competition varied seasonally and others depend on nesting status. However, male competition was never density-dependent (Table 2). Males were equally likely to be the recipients of physical competition at high and low male density (50 % of low density males and 42.5 % of high density males), and whether or not they were nest owners (47.5 % of nesting males and 43.6 % of non-nesting males), but were attacked more often late in the season than they were early in the season (Table 2a). Focal males were also 24 % more likely to initiate competition with other males if they were nest owners, but the initiation of competition did not vary with male density (32 % of low density and 34.5 % of high density males) or the time of season (Table 2a).

**Table 2:** Generalized estimating equations (A) and REML mixed models (B) investigating the factors that affected male competition (measured during *Focal Follows*)

A.				
**Behaviour**	**Fixed effect**	***Wald Chi-Square***	*df*	*P*
Competition received	Male Density	0.28	1	0.59
	**Day of Season**	**16.84**	**3**	**<0.01**
	Nesting Status	0.05	1	0.83
Competition initiated	Male Density	0.92	1	0.34
	Day of Season	6.61	3	0.08
	**Nesting Status**	**4.38**	**1**	**0.04**
B.				
**Behaviour**	**Fixed Effect**	***F***	***df***	***P***
Number of behaviours received	Male Density	0.43	1, 31.94	0.51
	**Day of Season**	**5.69**	**1, 27.51**	**0.02**
	Nesting Status	0.10	1, 109.9	0.76
Number of behaviours initiated	Male Density	0.01	1, 32.2	0.91
	**Day of Season**	**4.51**	**1, 25.23**	**0.04**
	Nesting Status	1.00	1, 114	0.32
Competition score	Male Density	0.21	1, 33.07	0.65
	Day of Season	0.08	1, 30.12	0.78
	**Nesting Status**	**8.85**	**1, 107.8**	**<0.01**
Number of competitors	Male Density	0.61	1, 31.8	0.44
	**Day of Season**	**8.81**	**1, 29.2**	**<0.01**
	Nesting Status	3.19	1, 106.4	0.08

GEEs investigated competition received (males receiving vs. not receiving aggression from non-focal males) and competition initiated (males initiating vs. not initiating competition with non-focal males), and REML mixed models investigated the sum number of behaviours initiated and received, how far competition escalated (the competition score), and the number of males physically competing. In the GEEs, we treated time as a categorical variable with four levels (dividing the season into 2 week blocks) because of lost degrees of freedom. In all other models, day of season was a continuous variable. Replicate pool was a random effect in all models and significant results are indicated in bold

**Fig. 3:**
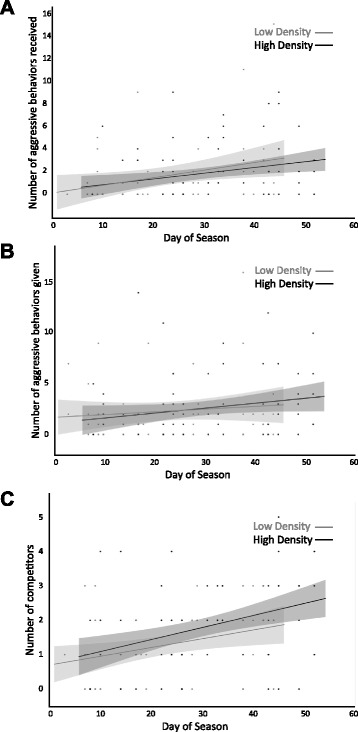
Seasonal patterns of aggression received (**a**), aggression given (**b**), and the number of competitors (**c**) across male densities. Represented are the linear regression lines and confidence intervals (*shadows*)

When males did initiate or receive competition from non-focal males (Table 2b), the number of behaviours observed was strongly seasonally-dependent, peaking late in the season (Fig. 3a). Males initiated 2.55 ± 0.38 competition interactions at high male density and 2.47 ± 0.51 at low male density, and received 1.69 ± 0.33 competition behaviours at high density and 2.04 ± 0.42 at low density. Nesting males initiated 2.81 ± 0.47 and received 1.79 ± 0.37 aggressive behaviours, and non-nesting males initiated 2.21 ± 0.40 and received 1.93 ± 0.32 aggressive behaviours. The intensity of competitive interactions varied with nesting status (Table 2b, Fig. 3b) but not density treatment (low = 1.51 ± 0.21, high = 1.63 ± 0.17) or time of season. Fights with nesting males were more intense (1.87 ± 0.18) than those with non-nesting males (1.27 ± 0.15). Finally, consistent with the finding that focal males initiated and received more aggressive behaviours late in the season (Table 2a), the number of males competing with the focal male also peaked late in the breeding season (Table 2b, Fig. 3c). The number of males physically competing with the focal male was independent of male density (low = 1.51 ± 0.20, high = 1.72 ± 0.17; Table 3b) and nesting status (nesting = 1.79 ± 0.17, non-nesting =1.44 ± 0.15), however.

**Table 3:** Results of REML mixed models investigating factors affecting the female mate search and mating decisions

**Behaviour**	**Fixed effect**	*F*	*df*	*P*
Number of males sampled	**Male Density**	**12.89**	**1, 21.1**	**<0.01**
	Day of Season	1.04	1, 22.62	0.32
Number of males with whom female initiated courtship	Male Density	0.94	1, 18.83	0.35
	Day of Season	0.29	1, 21.06	0.59
Number of female courtship initiation behaviours^a^	Male Density	0.98	1, 17.33	0.34
	Day of Season	0.72	1, 18.97	0.41
Number of male courtship behaviours^a^	Male Density	0.02	1, 14.54	0.90
	Day of Season	<0.01	1, 16.14	0.94
Acceptance score	Male Density	0.01	1, 18.19	0.92
	Day of Season	0.05	1, 22.93	0.82
Responsiveness (follows/lead)	Male Density	<0.01	1, 4.79	0.95
	Day of Season	0.29	1, 9.785	0.60
Chosen male red index	**Male Density**	**4.95**	**1, 10.60**	**0.02**
	**Day of Season**	**7.13**	**1, 10.35**	**0.01**
Chosen male red index rank	Male Density	<0.01	1, 15.08	0.96
	Day of Season	0.07	1, 17.12	0.81
Chosen male courtship vigour^a^	Male Density	0.45	1, 10.48	0.52
	Day of Season	0.03	1, 12.49	0.87
Chosen male courtship vigour rank	**Male Density**	**7.78**	**1, 15.92**	**0.01**
	Day of Season	0.02	1, 18.06	0.89

Significant results are indicated in bold

^a^indicates models in which behaviours were assessed per minute to account for trials ending upon nest entry

### Female experience

Male interactions with caged focal females during female experience trials consisted of approaches, zigzagging, biting (at the glass), and leading to the nest. We observed 28.59 ± 4.86 behaviours during each female experience trial (range 2 to 155). Females were courted by 2.29 ± 0.28 males during *Female Experience* trials at low male density and 3.12 ± 0.28 males at high male density (F_1, 17.88_ = 4.28, *p* = 0.053), but the sum number of courtship behaviours received per trial did not differ across densities (low = 14.24 ± 6.75, high =24.84 ± 6.65; F_1, 21.4_ = 1.22, *p* = 0.28). Although the difference in number of males courting females across treatments was just non-significant (*p* = 0.053) during the *Female Experience* trials, the pattern is consistent with a highly significant difference observed during the *Search and Choose* trials (below). Females observed 6.47 ± 1.32 male-male competitive interactions during female experience trials at low male density and 6.50 ± 1.15 at high male density. Consistent with results from the male *Focal Follows*, the sum number of competitive interactions did not differ across treatments (F_1, 10.74_ = 0.08, *p* = 0.78). Time of season did not influence the number of males courting females (F_1, 19.46_ = 0.14, *p* = 0.71), the sum number of courtship interactions with males (F_1, 22.87_ = 1.15, *p* = 0.29), or the number of competition behaviours observed by females during (F_1, 12.26_ = 0.34, *p* = 0.57).

### Search and choose trials

**Fig. 4:**
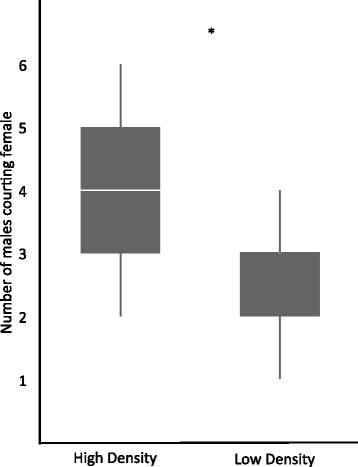
Number of males courting the searching female during *Search and Choose* trials at high and low male density. Boxes enclose the interquartile range (IQR) and horizontal bars represent the median. Whiskers extend to include the furthest point within 1.5x IQR from the box

We observed 92.97 ± 15.23 behaviours (range 12–460) during *Search and Choose* trials. Females undertook a restricted mate search, but sampled more males at high male density than they did at low male density (Table 3, Fig. 4; low = 2.52 ± 0.25, high = 3.84 ± 0.25). We included in “sampling” any observed interaction with a male that involved courtship (whether or not the males’ nest was complete) and/or visiting the male at the nest. This pattern was not driven by female initiation of courtship. Females initiated courtship 0.48 ± 0.09 times per minute with 2.71 ± 0.40 males at high density and 0.35 ± 0.89 times per minute with 2.17 ± 0.39 males at low density (Table 3). The number of courtship behaviours per minute did not vary with male density (Table 3). Neither the number of males sampled, the number of males with whom a female initiated courtship, the number of female initiation behaviours, nor the number of courtship behaviours observed were seasonally variable (Table 3).

To assess the motivation of females to mate, we looked at female responsiveness (follows/lead). We found no difference in responsiveness across treatments or time of season (Table 3). Females responded positively to 67 ± 13 % of male leads at high density and 66 ± 13 % of male leads at low density. Similarly, courtship events proceeded as far at high density as they did at low density (acceptance score did not depend on the density of males; Table 3). The mean acceptance score was 2.00 ± 0.38 at high density and 1.94 ± 0.39 at low density.

**Fig. 5:**
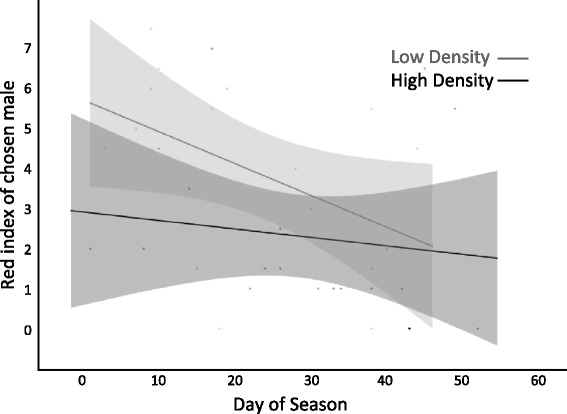
Red throat index of the chosen male at high and low male densities over the breeding season. Represented are the regression lines and confidence intervals (*shadows*)

Although female motivation to mate and acceptance of males did not differ depending on density of breeding males, the advertised quality of the males they ultimately pursued did. Because females did not always enter the nest of a male, and spawning was too rare to assess the decisions of females who spawned and did not spawn separately, we identified the “chosen” male as the male with whom the female proceeded furthest in the courtship process. In the case of ties, (for instance, a female might inspect the nests of two showing males) we looked to the number of behaviours to indicate whom the female was most interested in as a mate. It is not uncommon for spawning rates in British Columbian stickleback studies to be low (<20 %, RMT and JWB personal observation), making spawning a poor measure of mate choice in laboratory studies. The red throat indices of males chosen at low density were 76 % greater than males chosen at high density (Fig. 5, Table 3). Chosen males’ throat colour indices also peaked early in the season and declined over time. We also assessed the rank of a males’ red throat index relative to others in his replicate. We standardized these ranks to account for the difference in number of males across densities (such that ranks ranged from −3 to +3). The throat colour rank of males chosen at high and low breeding male density did not differ, suggesting females used a relative sampling strategy (Table 3).

Finally, behavioural observations suggested that males chosen as mates at high male density were particularly vigorous courters. We asked whether the males chosen at high male density performed more courtship behaviours per minute (chosen male courtship vigour) or were more vigorous courters than their pool-mates (chosen male courtship vigour rank, standardized to account for the difference in number of males across densities) in two REML mixed models (Table 3). The courtship vigour of chosen males was independent of male density and day of season (Table 3), however, males chosen as mates at high male density had a significantly higher courtship vigour rank than did those males chosen as mates at low density (Table 3). This suggests courtship vigour may be an important component of mate choice when many males compete for access to a single female mate.

## Discussion

Phenotypic plasticity in mate choice is enjoying a heyday of interest. Our current understanding, however, lacks integration across stages of mate choice, and empirical work assessing how female sampling strategies respond to environmental inputs is minimal. We assessed the density-dependence of early stages of mate choice, including the search for mates. In short, we found that pre-copulatory stages of mate choice were not modified in parallel as males and females responded to variation in mate availability. Sexual signalling was extremely plastic, including in response to male density (Fig. 1), but did not reflect the number of males with whom an individual physically competed nor the number of competitive behaviours a male experienced (at least after nests have been established, see below; Fig. 2). And, although females did search through more males at high male density than they did at low male density (as expected, suggesting plasticity in mate searching), they did not ultimately end up with more attractive males. Instead, we found that females were equally accepting of males at high and low density despite males at high density expressing less preferred signals. Without assessing the early stages of mate choice, this result would appear puzzling – why might females “choose” less attractive males at high density when low search costs afford them an opportunity to search for higher quality males? Because male density on the breeding grounds strongly influences male sexual signalling strategies, both the apparent quality of available mates and mate encounter rates may vary across densities.

Males attained greater throat colour at low male density than high male density, and signals peaked in late June, coincident with the majority of courtship and mate choice in the field (Table 1, Fig. 1). The density pattern is consistent with work in other stickleback populations [50] and with previous work in the Paxton Lake population [11]. Candolin [50] found that male throat colour honestly signals parental ability when competitors are present, but not when they are absent, and Tinghitella et al. [11] found that male signalling peaks mid-season at male-biased adult sex ratios, which both of our density treatments were. This was by design, and reflects the ecology of the system; females arrive on breeding territories after males have established nests. If male competition alters signalling before females arrive on the breeding grounds, this may facilitate females choosing better mates at high density because competition limits males’ dishonesty.

Why are males redder at low density? The pattern suggests one of two possibilities: 1) males may reduce their signalling effort at high density or 2) not be able to produce high intensity signals because of trade-offs with costly competition. In a number of systems, including *Gasterosteus* spp., males with exaggerated sexual signals receive fewer attacks and enjoy higher dominance status [51, 54]. So, reducing signalling effort may be unlikely to help males avoid costly competition in this system. It is possible that a combination of socially enforced costs of signalling and metabolically costly sexual signals may be responsible for lower average colour at high male density. Males may also be unable to produce high intensity signals at high male density because of trade-offs with competition. However, this explanation predicts greater or more intense competition at higher densities. We found no evidence for density-dependent male competition (Fig. 2). This contrasts with some previous work (e.g. [15, 28]), but is consistent with empirical work in European bitterlings and seed bugs in which male territorial aggression (and spawning disruption in the bitterlings) were rarer at high male density because territoriality and resource defence polygyny, respectively, broke down at high population densities [29, 31]. Moreover, when there are many male competitors, and therefore a lower chance of securing a mate, it may be more advantageous for males to engage in other strategies that increase fitness (like parental care), which were not measured here [19, 20]. Perhaps experimental design explains the divergence from theory. We surveyed male competition after males had established territories and built nests, so disagreements were already largely settled and there should be selection to rely on signalling rather than continue fighting (a dear enemy effect; [68, 69]).

Both *Female Experience* and *Search and Choose* trials support the finding that females sampled more males at high than low density, as expected, although the number of courtship behaviours they experienced was approximately equivalent across densities (Table 3, Fig. 4). Females were also equally as responsive to courtship and accepting of courting males across densities (Table 3). Moreover, males and females do not appear to switch sex roles nor do females initiate courtship more when competition for males is greater, as has been observed in two-spotted goby populations [12, 70].

That females sample more males at high male density suggests that mate search strategies are density-dependent. Despite the extended search, they ultimately pursue more attractive males at low male density because males express greater red throat colouration at low density overall. This could be consistent with a strategy in which females are searching for males who exceed a particular threshold (an absolute sampling strategy); given the greater expression of red throat colour at low densities, females find these males faster (sampling fewer potential mates) at low density. This is similar to findings in satin bowerbirds (*Ptilonorhynchus violaceus*), for instance, in which the loss of attractive, preferred mates from a particular region led females to expand their mate search [71]. However, chosen males differed in absolute throat colour, but not throat colour rank. In other words, successful males in high and low density treatments had similar ranking with respect to throat colour (chosen males at each treatment density were above the mean). This suggests instead that females are using relative search strategies. During the search for mates, they select males with the more developed sexual signals regardless of the position of those traits with respect to population means. Had we not assessed the early stages of mate choice, specifically plasticity in signalling, we might have wrongfully concluded instead that females are more discriminating at low male density.

Finally, careful observation of courtship at high and low male nesting densities suggested that courtship vigour may contribute to female choice at high density, but not low density. Although overall vigour did not differ across densities, the vigour rank of chosen males was higher at high density than at low (Table 3), suggesting that this particular trait contributes to female choice when males are plentiful. Courtship vigour may help females identify high quality mates when conspicuous colour signals were reduced [50]. It may also reflect male courtship strategies – at high density, if male colour is reduced, males may compensate with vigorous courting. There is little previous evidence that vigour is a trait preferred by female sticklebacks; in fact most studies have found that females do not prefer more vigorously courting males [62, 72, 73] and that vigorously courting males may be poor fathers [74]. This pattern deserves attention.

One limitation of this study is that by only releasing the non-focal female from the pool immediately prior to the Search and Choose trials, we altered not only the male density, but also the OSR across treatments (but only at this stage of the behavioural trials). Our rationale for doing so was that releasing both females would lead to female-female competition. The focus of our study was on mate search strategies, not female-female competition. This methodological detail makes it difficult to conclude definitively whether females altered their search patterns in response to their experience with the number/quality of males in the pool over the last hour (during *Focal Follows* and *Female Experience*), or in response to the more immediate change in female abundance. We think the latter is less-likely because the effects on outcome variables that were assessed in both *Female Experience* trials and *Search and Choose* trials (number of males courted by/sampled, number of courtship behaviors) were consistent with one another statistically.

## Conclusions

The manner in which females sample males has important implications for the strength of sexual selection. Our results agree with existing theory in that the opportunity for sexual selection is greater at higher male densities – females search through more males under those conditions, generating mating skew. However, to our knowledge, existing theory does not account for the manner in which variation in signalling interacts with changing search patterns. A more realistic picture of experience-dependent mate choice would benefit from integrating our understanding across stages of choice, particularly by incorporating plasticity in signalling.

## Additional file

Additional file 1: Table S1. Which describes the behaviours recorded during Male Focal Follows, Female Experience, and Search and Choose trials. (PDF 60 kb)

## Abbreviations

OSR: Operational Sex Ratio
REML: Restricted Maximum Likelihood
IQR: Interquartile Range
