# Supplementary material for: Females sample more males at high nesting densities, but ultimately obtain less attractive mates
Source: BMC Evol Biol. 2015 Sep 18;15:200. doi: 10.1186/s12862-015-0481-3 (PMC4575468; doi:10.1186/s12862-015-0481-3)
Supplement: Additional file 1: Table S1. — Which describes the behaviours recorded during Male Focal Follows, Female Experience, and Search and Choose trials. (PDF 60 kb) [file 12862_2015_481_MOESM1_ESM.pdf]

Additional file in support of: Tinghitella RM, Stehle C & Boughman JW. Females sample more males at high nesting densities, but ultimately obtain less attractive mates. *BMC Evolutionary Biology*.

Table S1. Behaviours recorded during *Male Focal Follows*, *Female Experi*ence, and *Search and Choose* trials indicative of female interest, courtship, and male competition.

**Behaviour Type**	**Behaviour**	**Description**
Female Interest	Approach	Movement towards male to within 2 cm
	Angle	Female's body at 45° incline
	Head-up	Swift motion into an "Angle"
	Follow	Trails male after a "Lead"
	Examine	Inspects nest following a "Show"
	Enter	Proceeds into male's nest following "Examine"
	Terminate courtship	Leaves vicinity of male during a courtship event
Male Courtship	Approach	Movement towards female to within 2 cm
	Bite	Male nips female with mouth
	Chase	Male swims behind female vigorously
	Zig-Zag	Quick left-right movements
	Dorsal Prick	Male sticks female with dorsal spines
	Lead	Directs female towards territory/nest
	Show	Identifies entry to nest in presence of female
	Rub	Male draws his body along dorsal side of the female during nest entry
	Terminate courtship	Leaves vicinity of female during a courtship event
Male Competition	Charge	Quick male movement towards male to within 2cm; usually at the edge of territory
	Bite	Male nips male with mouth
	Chase	Male swims behind male vigorously
	Interrupt	Male disturbs another males' courtship event by engaging in courtship with the focal female or competition with the courting male
	Mouth-wrestling	Two males lock jaws and tustle
	Nest Destruction	Male removes nesting material from another males' nest
